# Grade 3 Neuroendocrine Tumor (G3NET): An Unusual Tumor With A Histochemical Surprise

**DOI:** 10.7759/cureus.19103

**Published:** 2021-10-28

**Authors:** Sachi Singhal, Goonja Patel, Rohan B Singh, Rashmika Potdar

**Affiliations:** 1 Internal Medicine, Crozer-Chester Medical Center, Upland, USA; 2 School of Medicine, St. George's Univeristy, St. George, GRD; 3 Ophthalmology, Massachusetts Eye and Ear, Boston, USA; 4 Hematology and Medical Oncology, Alliance Cancer Specialists, Ridley Park, USA

**Keywords:** neuroendocrine neoplasms, oncology, ki67, g3net, chemotherapy

## Abstract

Given the rare occurrence and the indolent course of Neuroendocrine tumors (NETs), few epidemiological studies exist on these cancers. A small but interesting subset of these tumors are G3- Neuroendocrine Tumors. Grade 3 Neuroendocrine tumors (or G3-NETs) are unique in their intermediate prognosis. These grade discordant tumors lie in between high-grade poorly differentiated neuroendocrine carcinomas and grade 2 well-differentiated NETs, which have a worse and better prognosis than this rare entity, respectively. In this case report, we present a case of Grade 3 NET with an unusually high Ki67 index, diagnosed upon biopsy of metastasis visualized on imaging. Additionally, we review existing literature for characteristics, immunohistochemical markers, prognosis, and treatment modalities for the same.

## Introduction

Neuroendocrine neoplasms are a heterogenous group encompassing two fundamental groups of tumors: well-differentiated neoplasms with a low degree of proliferation, i.e, neuroendocrine tumors (or NETs), and poorly differentiated neoplasms with a high proliferative index, i.e. neuroendocrine carcinomas (or NECs). Given the rare occurrence and the indolent course of NETs, few epidemiological studies exist on these tumors. Dasari et al state the most recent numbers to be 6.98 NETs per 100,000 persons as of 2012 [1]. The 2019 WHO classification which classifies these tumors based on their Ki67 index and degree of differentiation, now includes a subset of these tumors, the G3-NETs. They are unique in their grade discordance. We present the case report of a 62-year-old gentleman who came in for shortness of breath and was found to have lesions in the liver on imaging that were eventually revealed to be Grade3 Neuroendocrine tumors of an unknown primary source, with an unusually high Ki67 index.

## Case presentation

A 62-year-old gentleman presented to the hospital with dizziness and weakness. He also had subjective shortness of breath on presentation with no increase in oxygen requirements. He denied any productive cough, hemoptysis, chest pain, recent sick contacts, travel, recent antibiotic use, and hospitalization at this time. He had a past medical history significant for Type 2 diabetes mellitus, hypertension, COPD on home oxygen, hyperlipidemia, and prostate cancer treated with radiation ten years ago. He was an active smoker (10 cigarettes a day), with a 25 pack-year smoking history. He was on albuterol HFA and budesonide-formoterol inhalers, amlodipine, atorvastatin, metformin, prednisone, and triameterene-hydrochlorthiazide before presentation to the ED. On clinical examination, he was afebrile, tachycardic (HR 105 bpm), breathing at 18 bpm, and his blood pressure was slightly elevated (148/94). His BMI was 35.02 kg/m2. Mild wheezing was noted bilaterally on his pulmonary exam. His abdomen was soft, non-tender with no appreciable organomegaly. No obvious cervical, axillary or inguinal lymphadenopathy was noted. His cardiac, neurological, and musculoskeletal examination was otherwise unremarkable. In the ED, he received 2 puffs of an albuterol rescue inhaler, which led to the resolution of his shortness of breath.

Blood investigations showed a white cell count of 9.26 x 10^3^/ uL, hemoglobin of 12.6 x 10^6^/ uL, and a platelet count of 240 x 10^6^/uL. His electrolytes were within normal limits at presentation, and blood glucose was 147 mg/dL. His EKG showed normal sinus rhythm.

**Figure 1:**
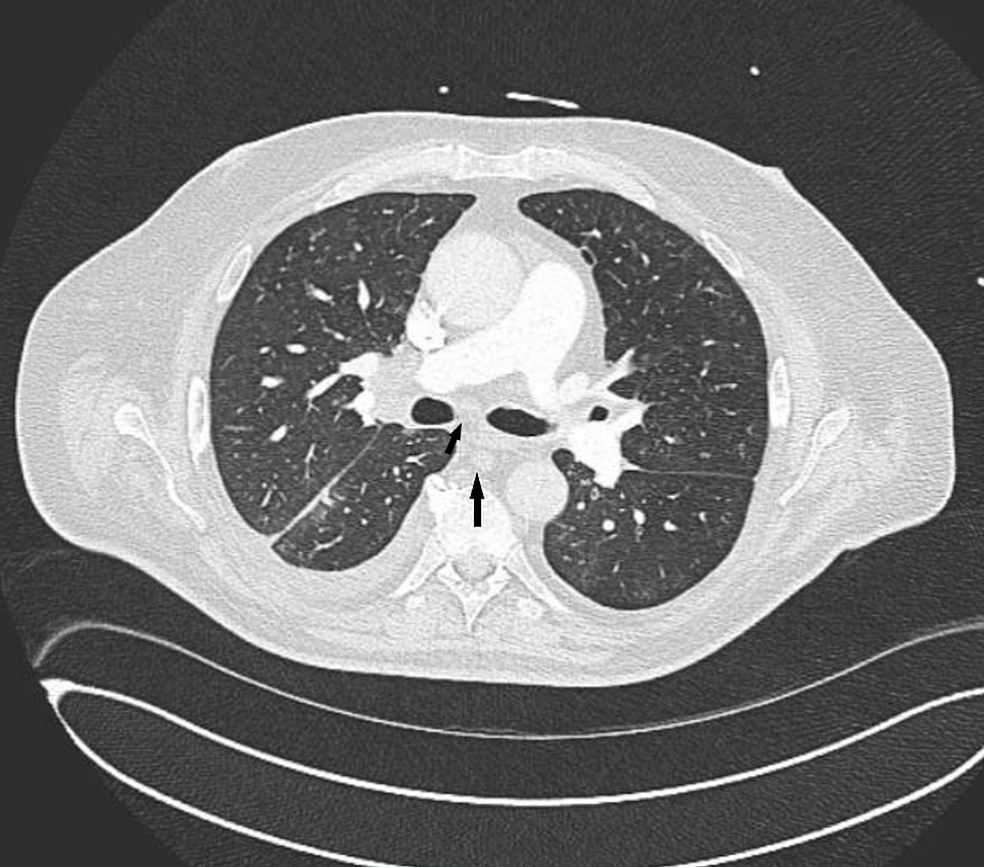
Axial view of CT-Angiogram on presentation, revealing hilar and infrahilar lymphadenopathy

**Figure 2:**
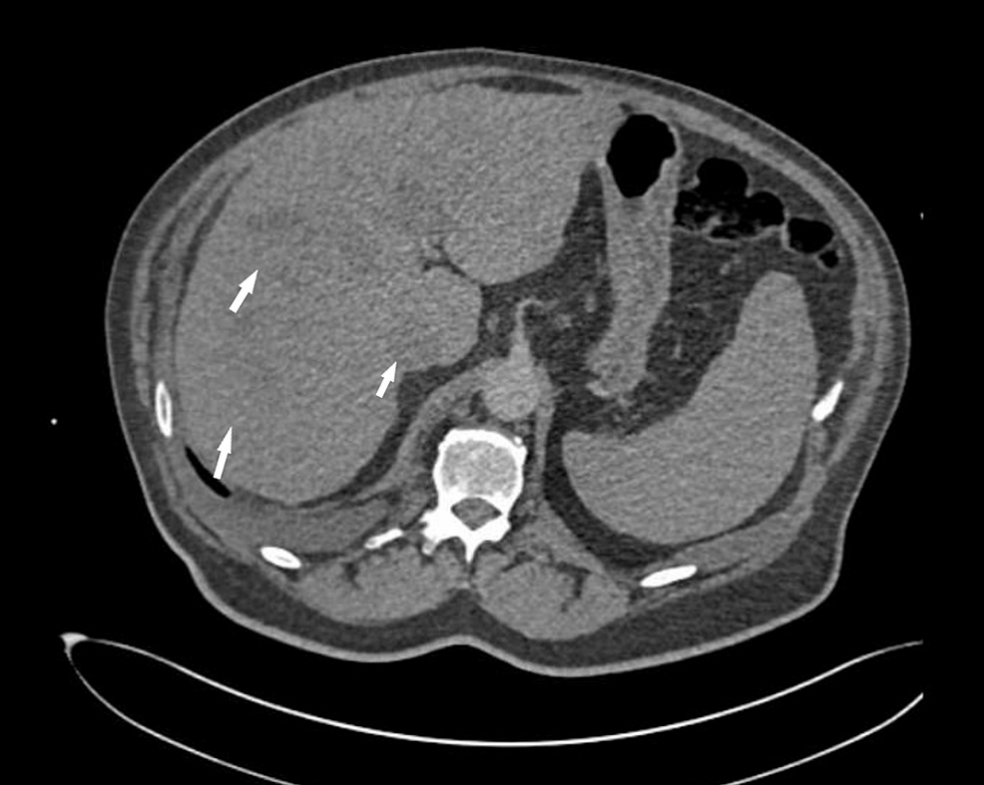
Axial view of CT-Angiogram from presentation showing several metastatic lesions in the liver

Given the patient’s active smoking history, subjective shortness of breath, obesity, and tachycardia on presentation, the decision for further imaging was made to rule out pulmonary embolus. Computed tomography-angiogram (CT-A) of chest and abdomen revealed adenopathy in the right hilum which measured up to 2.6 cm with small infra-hilar lymph nodes of 11-12 mm (Figure 1). There were multiple lesions throughout the liver (Figure 2), the largest being in the right inferior hepatic lobe measuring 2 cm. A preliminary diagnosis of liver metastasis was made. No primary cancer site was visible on this scan. Hematology/oncology recommended outpatient PET-CT for a detailed investigation of the extent of disease and identifying the primary site.

The patient admitted to never having a colonoscopy prior to this admission. To rule out the colon as the primary site, the decision to proceed with an in-patient colonoscopy was made, which showed two sessile polyps. They were 10-12mm in size and removed from sigmoid colon and transverse colon in the same procedure. Histopathology showed them to be tubular adenomas in nature.

Next, a liver biopsy was undertaken in this hospital stay to identify the type of cancer leading to metastasis. This biopsy showed cells arranged in large nests and sheets with areas of apoptosis and necrosis, as well as scattered mitotic figures; no small-cell carcinoma features were noted. Ki67 was positive in 80% of cells. There was 0% PDL-1 expression noted, and on immunohistochemical staining of this biopsied lesion, positivity for CK7, focal positivity of chromogranin was noted; strong positivity for GATA3, and weak patchy positivity for CDX2 was also noted. It was negative for TTF-1, PAX8, p40, NKX31, arginase, CD10, PR, and CK20. Taking into account these imaging, pathological and immunohistochemical findings, the diagnosis of well-differentiated neuroendocrine tumor with a high Ki67 index- G3NET was made, with the primary oncologic source unknown.

A detailed FDG-PET scan done after the hospital stay showed multiple osseous lesions and multiple liver metastases seen on prior imaging. The patient was then referred to a cancer-focused tertiary care institution and started on temozolomide-based targeted chemotherapy. He developed intermittent hematuria which was stable, and was being managed outpatient until three months later when he presented to our ED several times for urinary tract infection, pneumonia, and worsening shortness of breath. Of note, the patient continued to smoke one pack per day until one month prior to this hospital visit. He was admitted to the ICU for acute-on-chronic respiratory failure likely secondary to COPD exacerbation, worsening pneumonia and/or progression of cancer, and subsequently intubated. He had a STEMI and subsequently, cardiogenic shock requiring pressors. This was followed by ischemic hepatitis through his ICU stay, and his ejection fraction dropped to 20%. He also developed GI bleeding with acute blood loss anemia. Eventually, his family decided to shift to comfort-focused care after this long and protracted hospital stay, compassionately extubating him. The patient passed away the same day.

## Discussion

Neuroendocrine endoplasms (NEN) are divided into neuroendocrine carcinomas (NEC) and neuroendocrine tumors (NET) based on their molecular differences. Mutations in MEN1, DAXX, and ATRX are entity‐defining for well‐differentiated NETs, whereas NECs usually have TP53 or RB1 mutations. NETS are further divided into sub-classes based on their differentiation, grade, mitotic index, and Ki-67 index (as per 2019 WHO classification for the same). One of these subsets of neuroendocrine tumors called grade 3 neuroendocrine tumors or G3-NETs are high grade by proliferative or mitotic rate but remain well-differentiated on histology [2]. They are unique in their intermediate prognosis, mid-way between high-grade poorly differentiated neuroendocrine carcinomas, and grade 2 well-differentiated NETs.

Very limited data exists on the characteristics of Grade 3 NETs. A retrospective study from cases across eight European centers showed that the median age for these very rare tumors is 52 years. In about 65% of these tumors, the site of origin could be localized to the pancreas, while parts of the small and large intestine were very sparingly the site of origin. About 8% presented as cancer of unknown primary, or CUP, like our patient. The PRONET study showed 12.3% presented with metastasis of unknown origin. Most G3-NETs metastasized to the liver (78%), and some lymphatic, peritoneal, and bone metastasis were seen occasionally [3,4].

Most of the G3-NETs tested positive for synaptophysin, SRS, and chromogranin A+. FDG-PET was positive in about three-fourths of the cases. The median Ki67 index was 30%, which is in stark contrast to our patient, who had a Ki67 index of 80%. The very same study showed that almost all tumors with the Ki67 index of > 55% were poorly differentiated, which makes our case remarkable and worth reporting. This gentleman had a well-differentiated tumor on histology despite a very high mitotic index [3,4]. The biopsied lesion tested positive for chromogranin and synaptophysin, confirming the diagnosis of NET. Although the primary site of the tumor could not be identified despite PET and CT imaging, CDX2 positivity almost exclusively localizes this tumor to be of intestinal origin.

To the best of our knowledge, there is no standardized therapy established for G3-NET tumors. One line of thinking is that treatment modalities for G2-NET such as temozolomide-based chemotherapy or peptide receptor radiotherapy may be followed [5]. Sorbye et al demonstrated better response rates on these chemotherapeutic agents in neuroendocrine tumors with high Ki67 indices [6]. The median OS for G3-WDNET in another study was shown to be 41 months. It also showed well-differentiated G3-NETs, i.e WDNETs to have 0% complete or partial response to platin-based chemotherapy. Of note, not a single patient in this study had WDNET with Ki67 index >60% [7]. We plan to approach this highly unusual tumor aggressively, owing to the high mitotic index, and treat it like an NEC- with a platinum-etoposide regimen.

## Conclusions

We aim to highlight the highly unusual nature of an already rare tumor and tumor subtype, stressing the fact that neuroendocrine tumors overall, and Grade 3 NETs specifically are a highly heterogeneous group of rare tumors with sparse information on characteristics, presentations, and treatment modalities. This very fact makes clinical decisions extremely difficult. Our patient had a high Ki67 index despite being a well-differentiated tumor of unknown origin, which marks an overlap in the existing classification parameters of these tumors. We also aim to contribute the presentation and outcomes of this tumor in our patient to existing literature.

This case highlights that G3NETs are a very unique oncological diagnosis with an uncharacteristic mitotic index despite being well differentiated. Histopathology is critical to diagnosing, staging, and prognosticating this rare tumor, and guides its management despite scarce guidelines for the same. 

## References

[1] Dasari A, Shen C, Halperin D (2017). Trends in the incidence, prevalence, and survival outcomes in patients with neuroendocrine tumors in the United States. JAMA Oncol.

[2] Nagtegaal ID, Odze RD, Klimstra D (2020). The 2019 WHO classification of tumours of the digestive system. Histopathology.

[3] Heetfeld M, Chougnet CN, Olsen IH (2015). Characteristics and treatment of patients with G3 gastroenteropancreatic neuroendocrine neoplasms. Endocr Relat Cancer.

[4] Scoazec JY, Couvelard A, Monges G, Guyétant S, Bisot-Locard S, Parot X, Lepage C (2017). Professional practices and diagnostic issues in neuroendocrine tumour pathology: results of a prospective one-year survey among French pathologists (the PRONET Study). Neuroendocrinology.

[5] Rinke A, Gress TM (2017). Neuroendocrine Cancer, therapeutic strategies in G3 cancers. Digestion.

[6] Sorbye H, Welin S, Langer SW (2013). Predictive and prognostic factors for treatment and survival in 305 patients with advanced gastrointestinal neuroendocrine carcinoma (WHO G3): the NORDIC NEC study. Ann Oncol.

[7] Vélayoudom-Céphise FL, Duvillard P, Foucan L (2013). Are G3 ENETS neuroendocrine neoplasms heterogeneous?. Endocr Relat Cancer.

